# Endoplasmic Reticulum Sorting and Kinesin-1 Command the Targeting of Axonal GABA_B_ Receptors

**DOI:** 10.1371/journal.pone.0044168

**Published:** 2012-08-27

**Authors:** Viviana Valdés, José Ignacio Valenzuela, Daniela A. Salas, Matías Jaureguiberry-Bravo, Carolina Otero, Christina Thiede, Christoph F. Schmidt, Andrés Couve

**Affiliations:** 1 Biomedical Neuroscience Institute (BNI), Faculty of Medicine, Universidad de Chile, Santiago, Chile; 2 Program of Physiology and Biophysics, Institute of Biomedical Sciences (ICBM), Faculty of Medicine, Universidad de Chile, Santiago, Chile; 3 School of Biochemistry, Faculty of Biological Science, Universidad Andrés Bello, Santiago, Chile; 4 Faculty of Medicine, Pontificia Universidad Católica de Chile, Santiago, Chile; 5 Georg-August-Universität, Fakultät für Physik, Drittes Physikalisches Institut-Biophysik, Göttingen, Germany; Federal University of Rio de Janeiro, Brazil

## Abstract

In neuronal cells the intracellular trafficking machinery controls the availability of neurotransmitter receptors at the plasma membrane, which is a critical determinant of synaptic strength. Metabotropic γ amino-butyric acid (GABA) type B receptors (GABA_B_Rs) are neurotransmitter receptors that modulate synaptic transmission by mediating the slow and prolonged responses to GABA. GABA_B_Rs are obligatory heteromers constituted by two subunits, GABA_B_R1 and GABA_B_R2. GABA_B_R1a and GABA_B_R1b are the most abundant subunit variants. GABA_B_R1b is located in the somatodendritic domain whereas GABA_B_R1a is additionally targeted to the axon. Sushi domains located at the N-terminus of GABA_B_R1a constitute the only difference between both variants and are necessary and sufficient for axonal targeting. The precise targeting machinery and the organelles involved in sorting and transport have not been described. Here we demonstrate that GABA_B_Rs require the Golgi apparatus for plasma membrane delivery but that axonal sorting and targeting of GABA_B_R1a operate in a pre-Golgi compartment. In the axon GABA_B_R1a subunits are enriched in the endoplasmic reticulum (ER), and their dynamic behavior and colocalization with other secretory organelles like the ER-to-Golgi intermediate compartment (ERGIC) suggest that they employ a local secretory route. The transport of axonal GABA_B_R1a is microtubule-dependent and kinesin-1, a molecular motor of the kinesin family, determines axonal localization. Considering that progression of GABA_B_Rs through the secretory pathway is regulated by an ER retention motif our data contribute to understand the role of the axonal ER in non-canonical sorting and targeting of neurotransmitter receptors.

## Introduction

Polarized protein trafficking in the neuron is critical for synapse formation, synapse maintenance and the regulation of synaptic strength. In all eukaryotic cells the endomembrane trafficking system includes a forward biosynthetic route constituted by the endoplasmic reticulum (ER), the ER-Golgi intermediate compartment (ERGIC), the Golgi apparatus and post-Golgi vesicles, and a recycling-degradative route constituted by endosomes and lysosomes. The unique architecture and size of neurons does not necessarily imply that the structure/function relationship of these organelles and their contribution to the secretory process are different than in other cell types. However, their spatial arrangement and contribution to local processing may be specially adapted to the complexities of the neuronal morphology [1]. How the neuron orchestrates this highly compartmentalized trafficking is poorly understood. In particular, how the local distribution of secretory components in the neuron impinges on intracellular trafficking and availability of neurotransmitter receptors remains for the most part unexplored.

GABA is the main inhibitory neurotransmitter in the nervous system and the metabotropic GABA_B_Rs are obligatory heteromers composed of two related subunits, GABA_B_R1 and GABA_B_R2 (for a comprehensive review of GABA_B_R structure, function, localization and pathological implications see [2]). Both belong to family C of G protein-coupled receptors, and contain a large extracellular N-terminal domain, seven membrane-spanning domains and an intracellular C-terminal domain. GABA_B_Rs are expressed in neurons throughout the brain and spinal cord. They are mainly perisynaptic receptors located in gabaergic and glutamatergic presynaptic terminals and postsynaptic sites. GABA_B_R1 binds agonists with high affinity whereas GABA_B_R2 couples to G_αi_ establishing a transactivation mechanism between the two subunits. At presynaptic terminals GABA_B_Rs inhibit voltage gated Ca^2+^ channels thereby inhibiting synaptic vesicle fusion and neurotransmitter release. At postsynaptic sites they activate inwardly rectifying K^+^ channels hyperpolarizing the postsynaptic neuron. In addition, stimulation of GABA_B_Rs decreases the levels of cyclic AMP. GABA_B_Rs have been implicated in epilepsy, anxiety, stress, sleep disorders, nociception, depression, cognition and addictive mechanisms to drug abuse. The relevance of studying GABA_B_R availability is further supported by clinical observations that report the appearance of tolerance to GABA_B_R agonists, an inconvenient side effect to therapy.

GABA_B_R subunits are synthesized in the soma and glycosylated in the ER [3], [4]. The progression of GABA_B_Rs through the secretory pathway is regulated by an RXR-type sequence (RSRR) in the C-terminal domain of GABA_B_R1 that functions as an ER retention motif in the absence of GABA_B_R2 [5]. The ER retention motif is masked upon association to GABA_B_R2, and assembled GABA_B_Rs exit the ER as heteromers destined for the plasma membrane. Consistent with ER retention acting as a limiting step GABA_B_Rs are abundant within intracellular compartments, especially the ER [6].

**Figure 1:**
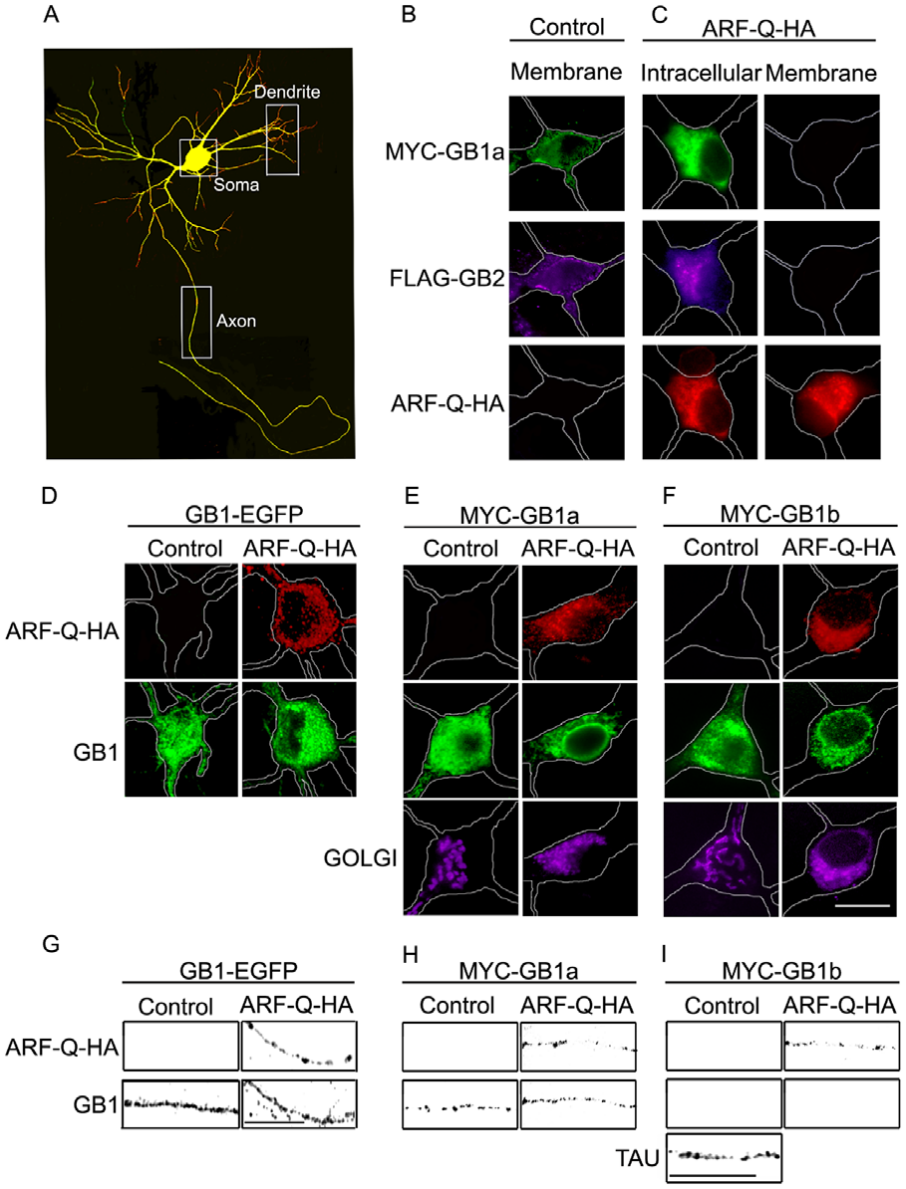
GABA_B_R1a is targeted to the axon even after blockade of Golgi export. (**A**) Hippocampal neurons were transfected with MYC-GABA_B_R1a and RFP, and processed for immunofluorescence under permeabilized conditions. MYC-GABA_B_R1a was detected with MYC antibodies (green) and RFP was detected without staining (red). Boxes indicate somatic, dendritic and axonal regions used throughout the study. (**B**) Hippocampal neurons were transfected with MYC-GABA_B_R1a and FLAG-GABA_B_R2 and processed for immunofluorescence under non-permeabilized conditions. MYC-GABA_B_R1a was detected with MYC antibodies (MYC-GB1a, green) and FLAG-GABA_B_R2 was detected with FLAG antibodies (FLAG-GB2, magenta) (representative image of n = 12 neurons). (**C**) Same as above for neurons transfected with MYC-GABA_B_R1a, FLAG-GABA_B_R2, and ARF1-Q71I-HA (ARF-Q-HA). ARF1-Q71I-HA was detected with HA antibodies (red). Neurons were labeled under permeabilized conditions to visualize intracellular GABA_B_R subunits or non-permeabilized conditions to evaluate their abundance at the plasma membrane (representative image of n = 28 neurons). (**D**) Hippocampal neurons from GABA_B_R1-EGFP mice in the absence (control) or presence of ARF1-Q71I-HA (ARF-Q-HA) and processed for immunofluorescence under permeabilized conditions. ARF1-Q71I-HA was detected with HA antibodies (ARF-Q-HA, red), GABA_B_R1-EGFP signal was amplified with EGFP antibodies (GB1, green) (representative image of n = 10 neurons). (**E**) Hippocampal neurons were transfected with MYC-GABA_B_R1a in the absence (control) or presence of ARF1-Q71I-HA (ARF-Q-HA) and processed for immunofluorescence under permeabilized conditions. ARF-Q-HA was detected with HA antibodies (red), MYC-GABA_B_R1a was detected with MYC antibodies (green), the Golgi apparatus was detected by expressing pEYFP-Golgi (magenta) (representative image of n = 18 neurons). **F**) Same as above for neurons transfected with MYC-GABA_B_R1b (representative image of n = 10 neurons). Scale bar for B-F represents 20 μm. **G–I**) Axons of hippocampal neurons under the experimental conditions of D-F (representative images of neurons examined above). Tau staining was used to visualize the axon when not labeled by MYC-GABA_B_R1b. Scale bar for G–I represents 20 μm.

GABA_B_R1a and GABA_B_R1b constitute the most abundant isoforms for GABA_B_R1. Heteromers containing GABA_B_R1a are axonal and somatodendritic whereas those containing GABA_B_R1b are exclusively located in the somatodendritic domain [7]. GABA_B_R1a and GABA_B_R1b mediate their different functions only as a result of their specific axonal or somatodendritic localization [7]. The sushi domains located at the N-terminus of GABA_B_R1a are necessary and sufficient for axonal targeting even in a GABA_B_R2 knock-out background [8]. However, the precise targeting machinery and the organelles involved in sorting and transport have not been described. Combining conventional optical microscopy and live-cell imaging using organelle reporters and trafficking blockers in cultured hippocampal neurons we describe a mechanism for GABA_B_R1a axonal localization based on pre-Golgi sorting and ER transport.

**Figure 2:**
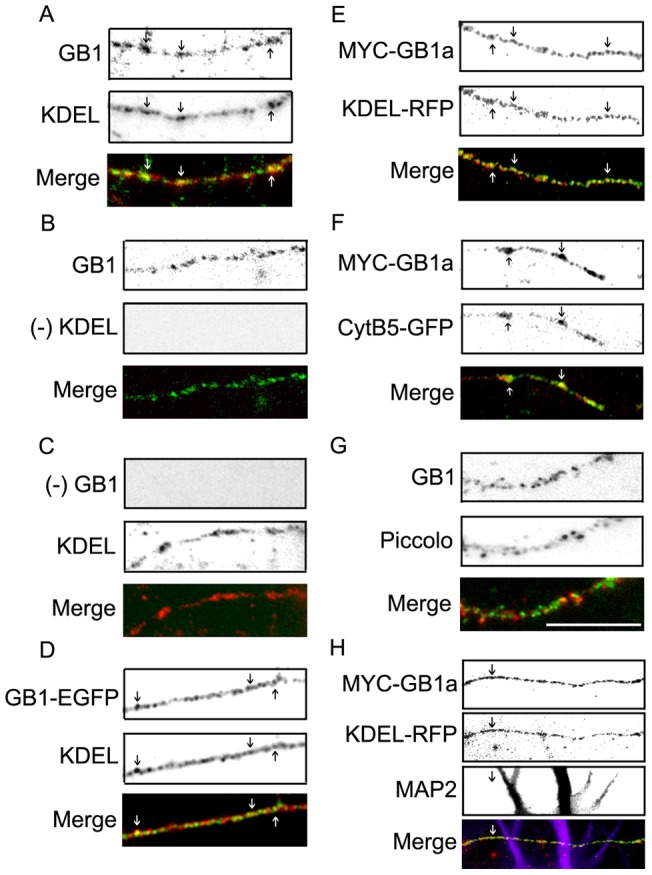
GABA_B_R1a colocalizes with the ER in axons. (**A**) Representative axon of hippocampal neurons processed for immunofluorescence under permeabilized conditions. GABA_B_R1 was detected with GABA_B_R1 antibodies (GB1, green) and the ER was detected with KDEL antibodies (KDEL, red). Merged images are shown on the bottom panel (representative image of n = 17 neurons). (**B**) Same as above excluding staining with primary KDEL antibodies. (**C**) Same as above excluding staining with primary GABA_B_R1 antibodies. (**D**) Representative axon of hippocampal neurons from GABA_B_R1-EGFP mice processed for immunofluorescence under permeabilized conditions. GABA_B_R1-EGFP signal was amplified with EGFP antibodies (GB1-EGFP, green) and the ER was detected with KDEL antibodies (KDEL, red). Merged images are shown on the bottom panel (representative image of n = 10 neurons). (**E**) Representative axon of hippocampal neurons transfected with MYC-GABA_B_R1a and KDEL-RFP and processed for immunofluorescence under permeabilized conditions. MYC-GABA_B_R1a was detected with MYC antibodies (MYC-GB1a, green), KDEL-RFP was visualized without staining (red). Merged images are shown on the bottom panel (representative image of n = 20 neurons). (**F**) Representative axon of hippocampal neurons transfected with MYC-GABA_B_R1a and cytochrome b5-EGFP (CytB5-GFP) and processed for immunofluorescence under permeabilized conditions. MYC-GABA_B_R1a was detected with MYC antibodies (MYC-GB1a, green), CytB5-GFP was visualized without staining (red). Merged images are shown on the bottom panel (representative image of n = 20 neurons). (**G**) Representative axon of hippocampal neurons processed for immunofluorescence under permeabilized conditions. GABA_B_R1a was detected with GABA_B_R1 antibodies (green) and Piccolo was detected with Piccolo antibodies (red). Merged images are shown on the bottom panel (representative image of n = 15 neurons). (**H**) Representative axon of hippocampal neurons transfected with MYC-GABA_B_R1a and KDEL-RFP and processed for immunofluorescence under permeabilized conditions. MYC-GABA_B_R1a was detected with MYC antibodies (MYC-GB1a, green), KDEL-RFP was visualized without staining (red) and MAP2 was detected with anti-MAP2 antibodies (MAP2, magenta). The axon is identified as a MAP2-negative projection. Merged images are shown on the bottom panel (representative image of n = 15 neurons). Scale bar for A-H represents 20 μm.

## Materials and Methods

### Animals

Adult pregnant female Sprague-Dawley rats were purchased from the Central Animal Facility at Universidad Católica de Chile and killed by asphyxia in a CO_2_ chamber according to the Guide for Care and Use of Laboratory Animals (The National Academy of Sciences, 1996). GABA_B_R1-EGFP mice were kindly provided by Bernhard Bettler (University of Basel, Switzerland). They correspond to transgenic animals for the GABA_B_R1-EGFP BAC in a homozygous knockout background for GABA_B_R1 as described previously [9], [10].

**Figure 3:**
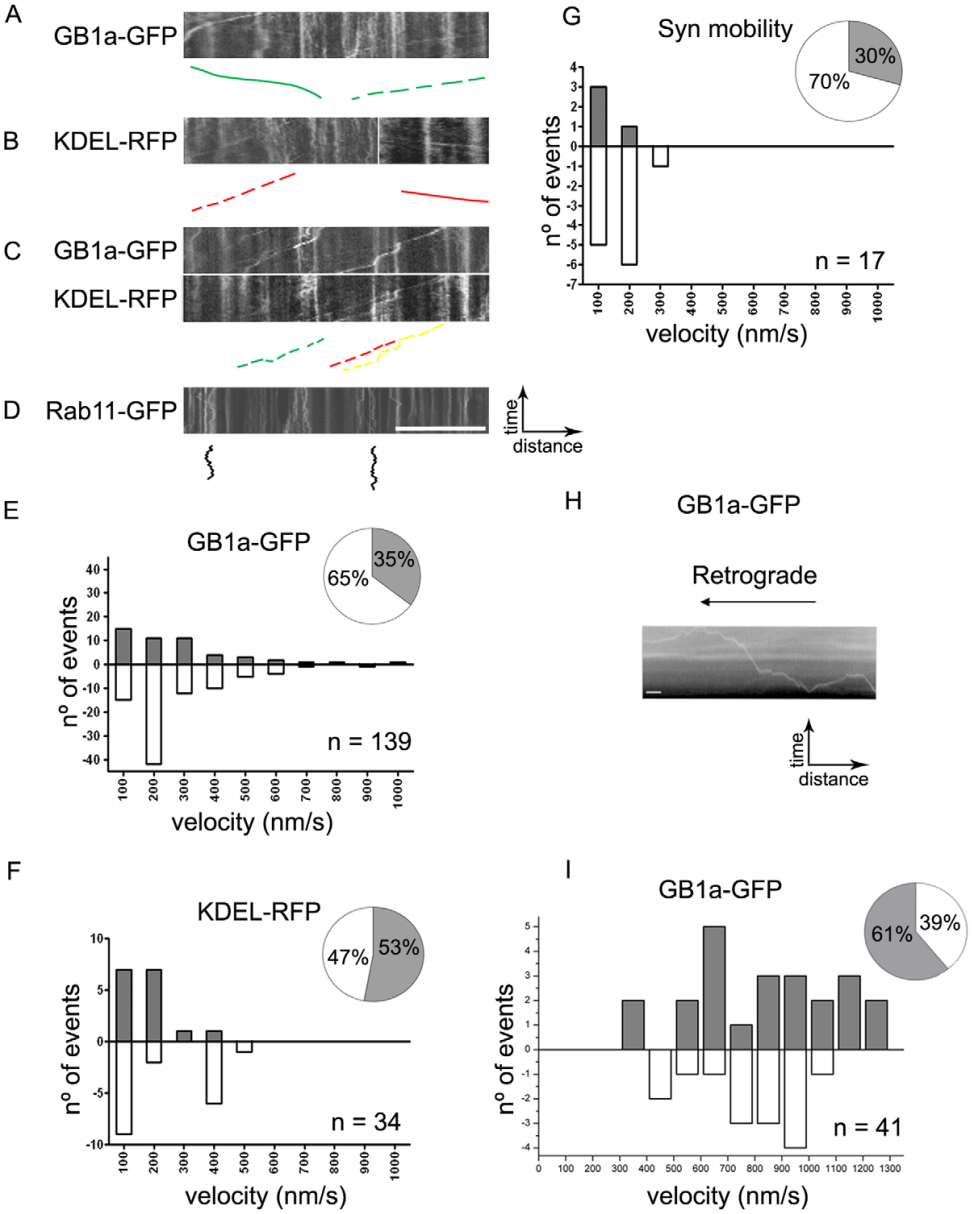
GABA_B_R1a and KDEL are mobile in axons. (**A**) Representative kymograph constructed from time series of axons of hippocampal neurons transfected with GABA_B_R1a-EGFP (GB1a-GFP). Color lines correspond to selected traces within the corresponding kymograph. Solid green line: anterograde mobility; dashed green line: retrograde mobility. (**B**) Same as above for KDEL-RFP. Solid red line: anterograde mobility; dashed red line: retrograde mobility. (**C**) Representative kymographs from time series of axons of hippocampal neurons transfected with GABA_B_R1a-EGFP and KDEL-RFP. Dashed green line: retrograde mobility of GABA_B_R1a-EGFP; dashed red line: retrograde mobility of KDEL-RFP; dashed yellow line: synchronous retrograde mobility of GABA_B_R1a-EGFP and KDEL-RFP. (**D**) Representative kymographs constructed from axons of hippocampal neurons transfected with Rab11-GFP. Solid black lines: short-range bidirectional mobility of Rab11-GFP (representative kymograph of n = 14 neurons). Images were acquired at 0.20–0.25 frames/s for a total of 120 s. Scale bar for A–D represents 20 μm. (**E–G**) Average velocity and directionality was quantified from the kymographs for GABA_B_R1a-EGFP, KDEL-RFP and synchronous mobility of GABA_B_R1a-EGFP and KDEL-RFP. Bar graphs represent the frequency distribution of velocities, anterograde transport (gray bars), retrograde transport (white bars). Pie charts represent fractions of anterograde (gray) and retrograde transport (white). Average velocities and direction were obtained from 17–139 moving puncta from a total of 42 neurons from at least three independent culture preparations. (**H**) Representative kymograph from time series of axons of hippocampal neurons transfected with GABA_B_R1a-EGFP imaged by TIRF microscopy at 2 frames/s. Scale bars represent 10 s and 3 µm. (**I**) Bar graph represents the frequency distribution of velocities: anterograde (gray bars), retrograde (white bars). Pie chart represents fractions of anterograde (gray) and retrograde transport (white). Average velocities and direction were obtained from 41 moving puncta from at least three independent culture preparations.

### Cell lines, neuronal cultures and transfection

COS-7 cells were maintained and transfected as described previously [3] using a Gene Pulser Xcell (BioRad). Primary hippocampal neurons were cultured from E18 rats or E18 GABA_B_R1-EGFP transgenic mice according to established procedures [11] and transfected by Ca^2+^ phosphate at 14–18 days *in vitro* (div) [12]. All transfected neurons, except for those in Fig. S2, were analyzed 1 day post transfection (dpt). Transfected neurons for Fig. S2 were analyzed between 1–5 dpt.

**Figure 4:**
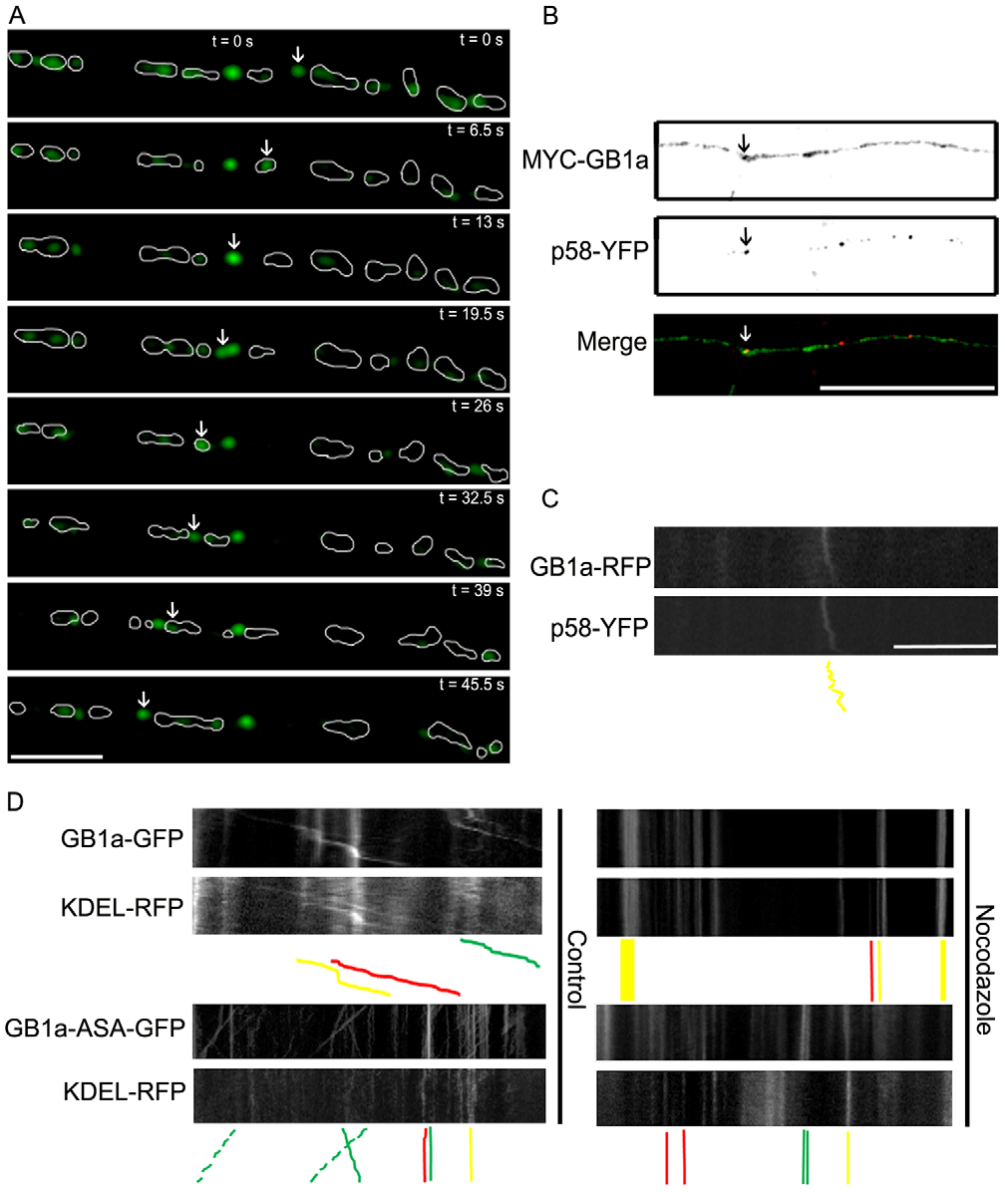
GABA_B_R1a cycles between KDEL compartments in axons. (**A**) Representative axon of hippocampal neurons transfected with GABA_B_R1a-EGFP and KDEL-RFP, and visualized live. The area originally occupied by the ER (KDEL-RFP) in each time frame was outlined in white. The arrows show a GABA_B_R1a-EGFP puncta that exits and enters different ER compartments within the observation period (45.5 s) (representative time-lapse sequence of n = 10 neurons). Scale bar represents 20 μm. (**B**) Representative axon of hippocampal neurons transfected with MYC-GABA_B_R1a (MYC-GB1a) and p58-YFP, and processed for immunofluorescence under permeabilized conditions. MYC-GABA_B_R1a was detected with MYC antibodies (green); p58-YFP was visualized without staining (red). Merged images are shown on the bottom panel (representative image of n = 23 neurons). Scale bar represents 20 μm. (**C**) Kymographs were constructed from time series of axons of hippocampal neurons transfected with GABA_B_R1a-RFP (GB1a-RFP) and p58-YFP. Solid yellow lines: synchronous transport of GABA_B_R1a-RFP and p58-YFP (representative kymograph of n = 22 neurons). Scale bar for C-D represents 20 μm. (**D**) Kymographs were constructed from time series of axons of hippocampal neurons under control conditions or treated with nocodazole after transfection with GABA_B_R1a-EGFP (GB1a-GFP) or GABA_B_R1a-ASA-EGFP (GB1a-ASA-GFP) and KDEL-RFP. Top right panels: accumulated GABA_B_R1a-EGFP and KDEL-RFP in static puncta (solid yellow lines). Bottom right panels: accumulated GABA_B_R1a-ASA-EGFP and KDEL-RFP in different static puncta (solid green and red lines) (representative kymograph of n = 21 neurons).

### Reagents and DNA plasmids

Nocodazole was purchased from Sigma (St. Louis, MO). MYC-GABA_B_R1, FLAG-GABA_B_R2, HA-GABA_B_R2, MYC-GABA_B_R1-AA-ASA, MYC-GABA_B_R1-ΔC in pRK5 have been described previously and contain epitope tags on the extracellular N-terminal domains [3], [13]–[15]. MYC-GABA_B_R1-AA-ASA contains point mutations that replace two leucine residues at a di-leucine motif and two arginine residues at the ER retention motif (RSRR) by alanines. MYC-GABA_B_R1-ΔC lacks the complete C-terminal domain. Both mutants escape the ER and traffic to the cell surface in the absence of GABA_B_R2. GABA_B_R1a-EGFP, GABA_B_R2-EGFP and GABA_B_R1a-monomeric red fluorescent protein have also been described previously and contain the fluorescent proteins attached to the intracellular C-terminal domain [4]. pDsRed-C1 (RFP), pEYFP-Golgi, pEYFP-ER and pDsRed2-ER (KDEL-RFP) were obtained from Clontech (Mountain View, CA). Kif5C-RFP-DN was kindly provided by S. Kindler and H.J. Kreienkamp (Institut für Humangenetik, Universitätsklinikum Hamburg-Eppendorf, Hamburg, Germany) and corresponds to amino acids 678–955 of KIF5C (NM_001107730) also referred to as DN2 by Falley and collaborators [16]. ARF1-Q71I-HA was kindly provided by O. Jeyifous (University of Chicago, Chicago, IL), Rab11-GFP was kindly provided by F. Bronfman (Pontificia Universidad Católica de Chile), p58-YFP was kindly provided by J. Lippincott-Schwartz (National Institutes of Health, Bethesda, MD). Cytochrome b5-EGFP was kindly provided by C. Hetz (Universidad de Chile). All manipulations and fidelity of DNA constructs were verified by sequencing.

**Figure 5:**
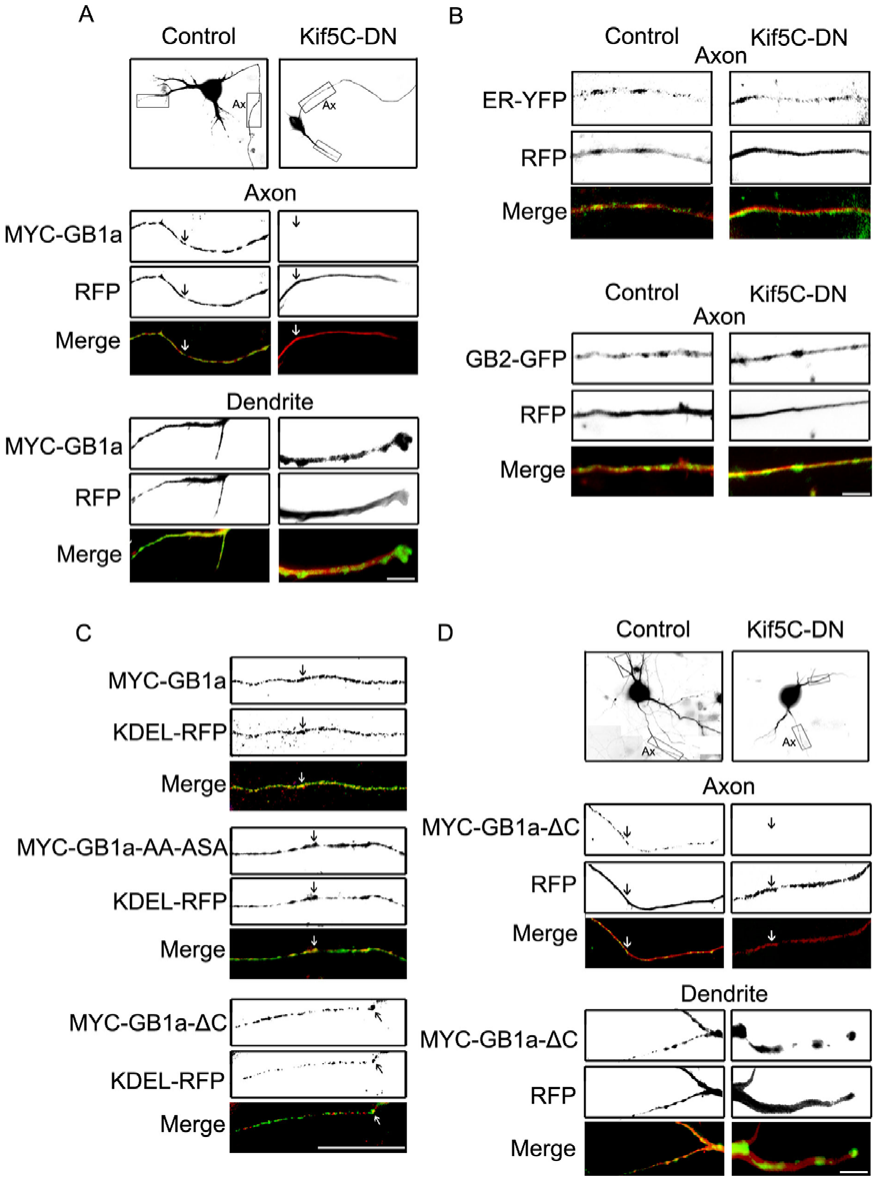
Axonal targeting of GABA_B_R1a is kinesin-1 dependent and C-terminal domain independent. (**A**) Hippocampal neurons were transfected with MYC-GABA_B_R1a and RFP (left, representative image of n = 12 neurons) or MYC-GABA_B_R1a and Kif5C-RFP-DN, a dominant negative version of kinesin-1 (Kif5C-DN, right, representative image of n = 13 neurons). The neuronal volume filled by RFP or Kif5C-RFP-DN is shown. Boxes in top panels correspond to regions of axons (Ax) and dendrites (unlabelled) magnified below. MYC-GABA_B_R1a (green) and RFP or Kif5C-RFP-DN (red). Merged images are shown on the bottom panel. Scale bar represents 40 μm. (**B**) Top panels: representative axon of hippocampal neurons transfected with pEYFP-ER (ER-YFP) and RFP or pEYFP-ER and Kif5C-RFP-DN. Markers were visualized without staining. Merged images are shown on the bottom panel (representative image of n = 15 neurons). Bottom panels: representative axon of hippocampal neurons transfected with GABA_B_R2-EGFP (GB2-GFP) and RFP or GABA_B_R2-EGFP and Kif5C-RFP-DN. Markers were visualized without staining. Merged images are shown on the bottom panel (representative image of n = 15 neurons). Scale bar for represents 40 μm. (**C**) Representative axons of hippocampal neurons transfected with KDEL-RFP and MYC-GABA_B_R1a, or MYC-GABA_B_R1a-AA-ASA, or MYC-GABA_B_R1a-ΔC, and processed for immunofluorescence under permeabilized conditions. MYC-GABA_B_R1a versions were detected with MYC antibodies (MYC-GB1a, MYC-GB1a-AA-ASA, MYC-GB1a-ΔC); KDEL-RFP was visualized without staining (representative images of n = 20, 9 and 10 neurons respectively). Merged images are shown on the bottom panels. Scale bar represents 20 μm. (**D**) Hippocampal neurons were transfected with MYC-GABA_B_R1a-ΔC and RFP (left, representative image of n = 12 neurons) or MYC-GABA_B_R1a-ΔC and Kif5C-RFP-DN (Kif5C-DN, right, representative image of n = 9 neurons). The neuronal volume filled by RFP or Kif5C-RFP-DN is shown. Boxes in top panels correspond to regions of axons (Ax) and dendrites (unlabelled) magnified below. MYC-GABA_B_R1a-ΔC (green) and RFP or Kif5C-RFP-DN (red). Merged images are shown on the bottom panel. Scale bar represents 40 μm.

### Antibodies

GABA_B_R1 antibodies (which recognize GABA_B_R1a and GABA_B_R1b) were raised against the intracellular C-terminal domain in rabbits and affinity purified. GABA_B_R1 antibodies specifically recognize MYC-GABA_B_R1a in transfected COS cells and detect the predicted doublet corresponding to GABA_B_R1a and GABA_B_R1b in crude brain membranes ( Fig. S1). Microtubule-associated protein 2 (MAP2) antibodies were purchased from Chemicon (Temecula, CA). Piccolo antibodies were kindly provided by ED. Gundelfinger and WD. Altrock (Leibniz Institute for Neurobiology, Magdeburg Germany). KDEL antibodies (directed against a 6-residue peptide (SEKDEL) of the rat Grp78 protein) and cis-Golgi matrix protein 130 (GM130) were purchased from StressGen (Ann Arbor, MI). MYC antibodies were purchased from Sigma (St. Louis, Missouri). Influenza A Virus epitope (HA) antibodies were purchased from Roche (Indianapolis, IN). Anti-GFP antibodies (ab6556) were purchased from Abcam (Cambridge, UK). Secondary anti-mouse, anti-rabbit, anti-guinea pig or anti-chicken antibodies conjugated to Texas Red (TR), tetramethyl rhodamine isothiocyanate (TRITC), fluorescein isothiocyanate (FITC) or cyanine 3 (Cy3) were purchased from Jackson Immuno Research Laboratories (West Grove, PA).

### Immunofluorescence, image capture, image processing and time-lapse microscopy

Immunofluorescence was performed as described previously under non-permeabilized or permeabilized conditions [4]. Depending on the type of experiment axons were identified by the presence of a positive marker (Tau), the absence of a negative marker (MAP2), or by morphological criteria that included: longer projection, constant diameter and right angle branching. Image-processing routines were developed in our laboratory based on of Interactive Data Language (IDL) (ITT, Boulder, CO), including routines for segmentation [4]. Live-cell imaging was performed in a 23°C equilibrated microscopy suite. Images were obtained using an Olympus BX61WI upright microscope and an Olympus disk-scanning unit. Consecutive frames were acquired over a period of ∼120 s. Kymographs were constructed from an axonal segment 20 µm from the soma using ImageJ from three-pixel-wide axonal traces. The axon-to-dendrite (A:D) ratio of MYC-GABA_B_R1a was determined using ImageJ according to a procedure based on Biermann *et.* *al*., 2010 and modified [8]. Briefly, one-pixel-wide lines were traced along the initial 150 µm of axons and dendrites in images labeled with soluble RFP or Kif5C-RFP-DN. No measurements were carried out beyond 150 µm to prevent artifacts due to axonal length differences between RFP or Kif5C-RFP-DN transfected neurons. Average pixel intensities of MYC-GABA_B_R1a were determined along the traced lines, background was subtracted and the data was used to determine the A:D ratio. Criteria for cell selection included even distribution of RFP or Kif5C-RFP-DN in axons and dendrites, and cells expressing constructs at very high levels were excluded from the analysis. 9–12 neurons from at least two independent culture preparations were analyzed for each condition. Images of axons from GABA_B_R1-EGFP mice were acquired using an Olympus FV-1000 confocal microscope. Anti-GFP antibodies were used to amplify the weak axonal signal of GABA_B_R1-EGFP expressed at physiological levels.

### Total Internal Reflection Fluorescence (TIRF) microscopy

For TIRF microscopy 14–18 div hippocampal neurons were transfected with GABA_B_R1a-EGFP and motility in axons was analyzed 1 dpt. TIRF was carried out on a custom-built TIRF microscope setup: Two lasers (473 nm and 532 nm, both 30 mW, Viasho, USA) were used to excite the fluorophores (GFP and RFP). The lasers were expanded and coupled via a multi-beam splitter (z474/488/532/635rpc, Chroma, USA) off-axis into the oil-immersion objective (Nikon, SFluor 100x, 1.49) to obtain TIRF illumination. The emitted fluorescent light was split in the GFP and RFP signals using a dichroic mirror (525/50, Chroma, USA) then passed trough bandpass filters (530/50 for GFP and 605/70 for TMR, both Chroma, USA) and finally directed via mirrors to separate areas on the chip of a frame transfer CCD camera (Cascasde:512B, Roper Scientific, USA). The CCD camera was controlled via WinSpec32 (Princeton Instruments, USA). The penetration depth of TIRF was 147 nm. Digital images were taken at a frame rate of 2 frames/s and were subsequently analyzed for velocity and direction using kymographs generated with a custom-written LabView (National Instruments, USA) routine. Kymographs were analyzed for velocity and direction by fitting lines to the segments of a trace judged by eye. Stalls were not taken into account.

## Results

### The delivery of GABA_B_Rs to the plasma membrane is Golgi-dependent but axonal targeting is not

First we carried out a control experiment to validate the use of overexpression of recombinant GABA_B_R subunits as a strategy to study receptor trafficking. Cultured hippocampal neurons were transfected with MYC-GABA_B_R1a and the distribution of the subunit at the plasma membrane or in intracellular compartments was evaluated by immunostaining 1–5 dpt. MYC-GABA_B_R1a was retained in intracellular compartments in the cell body and axons up to 5 dpt in the absence of recombinant GABA_B_R2 expression (Fig. S2). In contrast, GABA_B_R1a was readily detectable at the cell surface at 2 dpt upon co-transfection with GABA_B_R2 (Fig. S2, right column). These experiments indicate that the trafficking properties of recombinant receptors mimic the situation of the native subunits, and that the trafficking of recombinant receptors is not affected by the endogenous subunits. More importantly, they demonstrate that our experiments using transfection of recombinant GABA_B_R1 subunits exclusively examine their intracellular population.

To determine whether GABA_B_Rs employ a Golgi-dependent intracellular trafficking route in neurons, primary cultures of hippocampal neurons were transfected with MYC-GABA_B_R1a and FLAG-GABA_B_R2 in the absence or presence of ARF1-Q71I-HA, a constitutively active ARF1 mutant that prevents export from the Golgi apparatus [17]. 1 dpt the distribution of GABA_B_Rs at the plasma membrane and in intracellular compartments was evaluated by immunostaining under non-permeabilized or permeabilized conditions. We examined somatic or axonal domains as shown in the schematic neuron (Fig. 1A). As reported previously co-transfection of MYC-GABA_B_R1a and FLAG-GABA_B_R2 resulted in a robust localization of both subunits at the cell surface (Fig. 1B). In contrast, ARF1-Q71I-HA blocked the appearance of both subunits at the plasma membrane and produced accumulation in intracellular compartments (Fig. 1C). These results indicate that the Golgi apparatus is necessary for the delivery of GABA_B_Rs to the plasma membrane in hippocampal neurons.

GABA_B_R1a is targeted to the axon in hippocampal neurons [7], [8]. Thus, we determined whether axonal targeting was also Golgi dependent. First we used cultured hippocampal neurons of transgenic mice that express GABA_B_R1-EGFP under the control of an endogenous promoter [10]. This experimental model combines the advantages of a nearly physiological scenario and ease of detection. GABA_B_R1 localized to the axon in control conditions (Figs. 1D and 1G, left panel). Importantly, GABA_B_R1 was still targeted to the axon in the presence of ARF1-Q71I-HA (Figs. 1D and 1G, right panels). According to previous reports the predominant axonal variant of GABA_B_R1 corresponds to GABA_B_R1a [7], [8]. Therefore, these data are compatible with the idea that GABA_B_R1a is sorted and targeted to the axon at or prior to the Golgi apparatus.

Next we used recombinant receptors to directly compare the axonal targeting of GABA_B_R1a and GABA_B_R1b, and their Golgi dependence. Neurons were transfected with MYC-GABA_B_R1a or MYC-GABA_B_R1b in the absence or presence of ARF1-Q71I-HA. Consistent with previous reports, GABA_B_R1a but not GABA_B_R1b was predominantly targeted to the axon in hippocampal neurons (Figs. 1E-1I, control panels). Importantly, recombinant GABA_B_R1a was still targeted to the axon in the presence of ARF1-Q71I-HA (Figs. 1E and 1H). In contrast, GABA_B_R1b was absent from the axon under all the conditions examined (Figs. 1F and 1I). These findings indicate that axonal targeting is specific to GABA_B_R1a. In addition, they demonstrate that the sorting and targeting of GABA_B_R1a to the axon occurs at or prior to the Golgi stage, and therefore suggest a non-conventional modality. They also indicate that axonal targeting of GABA_B_R1a upon ARF1-Q71I-HA expression is not produced by an overload of the early secretory pathway because the effect was not observed for GABA_B_R1b, a conclusion further supported by the results in lower expressing transgenic neurons.

### GABA_B_R1a is targeted and transported within the axonal ER

ER resident proteins and components of the protein folding and export machineries localize to the axon [18]–[20]. Thus, to determine the intracellular localization of axonal GABA_B_R1a neurons were immunostained with antibodies against the GABA_B_R1 subunit and an antibody against the SEKDEL sequence of the rat ER protein Grp78, including a conserved motif present in luminal ER resident proteins responsible for retrieval from the Golgi apparatus [21]. Endogenous GABA_B_R1 colocalized with the ER in axons (Fig. 2A, arrows). Control labeling without primary antibodies confirmed the specificity of the signal (Fig. 2B and 2C). In transgenic mouse neurons GABA_B_R1-EGFP also colocalized with the ER (Fig. 2D, arrows). Importantly, colocalization was still observed upon overexpression of MYC-GABA_B_R1a and KDEL-RFP, a fluorescent probe widely used for ER visualization that contains the ER targeting sequence of calreticulin and the ER retrieval sequence KDEL (Fig. 2E, arrows). In addition, colocalization was observed with cytochrome b5-EGFP, another fluorescent ER probe (Fig. 2F). The colocalization between GABA_B_R1a and ER markers was specific because Piccolo, a marker for dense core vesicles and synapses [22] showed a markedly different axonal localization (Fig. 2G). Staining with MAP2, an exclusive dendritic marker confirmed the co-distribution of GABA_B_R1a and the ER occurs in the axon (Fig. 2H). These results demonstrate that GABA_B_R1a is enriched in the axonal ER.

To establish whether the ER functions as a transport organelle for GABA_B_R1a we examined the dynamic behavior of fluorescent versions of GABA_B_R1a and the ER in axons of live hippocampal neurons. Discrete GABA_B_R1a-EGFP and KDEL-RFP puncta were distributed along the axons. While the majority of puncta remained static or showed very limited lateral displacement over the examined period, a subset displayed continuous, long-range mobility (imaged at 0.20–0.25 frames/s for a total of ∼120 s, Figs. 3A and 3B). GABA_B_R1a-EGFP and KDEL-RFP moved bidirectionally, with a moderate retrograde bias, and with modal speeds of 100–200 nm/s (Figs. 3E and 3F). Significantly, some GABA_B_R1a-EGFP and KDEL-RFP puncta moved in synchrony, with similar speed and retrograde predominance (Figs. 3C and 3G). Puncta containing Rab11-GFP, a recycling endosome marker [23], also localized to axons but showed a different dynamic pattern characterized by rapid direction changes and lower overall displacement. We used TIRF microscopy and higher temporal resolution (2 frames/s) to determine the instant velocity of GABA_B_R1a-EGFP more accurately in hippocampal neurons. Mean anterograde and retrograde instant velocities were comparable (751.70±33.20 and 877.73±67.59 nm/s respectively) (Figs. 3H and 3I). These values fit conventional kinesin velocities [24] and their slight increase above panels A-G most likely result from excluding stalls in the analysis of our higher temporal resolution imaging.

A proportion of GABA_B_R1a-EGFP and KDEL-RFP puncta moved independently from each other. This may indicate that a fraction of GABA_B_R1a-EGFP is transported in a different secretory organelle or that the axonal ER compartment is capable of dynamically segregating cargo. To discriminate between these possibilities we carried out a series of complementary experiments. First we analyzed time-lapse microscopy sequences individually. Interestingly, GABA_B_R1a-EGFP puncta that initially colocalized with the ER sometimes separated from the organelle, remained segregated for a few frames and fused again with a pre-existing ER compartment (Fig. 4A). It is well known that ER cargo recycles between the ER and the ERGIC using export/retrieval motifs such as the RXR-type sequence present in GABA_B_R1a [25]. To determine whether the segregated GABA_B_R1a puncta resided temporarily in the ERGIC, we first visualized the axonal distribution of MYC-GABA_B_R1a and p58-YFP, an established marker of the ERGIC [26], in fixed cells. Sparse ERGIC puncta were observed in axons and a subset of them colocalized with MYC-GABA_B_R1a (Fig. 4B). Additionally, live-cell imaging was carried out in neurons transfected with GABA_B_R1a-RFP and p58-YFP. A small fraction of GABA_B_R1a-RFP displayed synchronous motility with p58-YFP in axons (Fig. 4C). Taking into account that intra-ER mobility is microtubule dependent but short-range ER to ERGIC transport is not [27], we reasoned that the enrichment of GABA_B_R1a in the ER should increase upon destabilization of microtubules. Consistent with this prediction, nocodazole blocked the mobility of GABA_B_R1a-EGFP puncta and the subunits accumulated in a KDEL-RFP compartment (Fig. 4D, top right panels). As expected, a mutant GABA_B_R1a subunit that is not retained in the ER (GABA_B_R1a-ASA-EGFP) accumulated in a different compartment after nocodazole treatment (Fig. 4D, bottom right panels). These observations suggest that axonal GABA_B_R1a is targeted and transported to the axon within the ER, and possibly engages in a local export/retrieval mechanism between the ER and the ERGIC.

### Kinesin-1 contributes to the axonal localization of GABA_B_R1a

Since kinesin-1, an axonal biased molecular motor [28], [29], colocalizes with GABA_B_R1 in neurons, and associates to the subunit in fractionation and coimmunoprecipitation assays [30] we evaluated its contribution to axonal GABA_B_R1a targeting. To examine the axonal localization of MYC-GABA_B_R1a we determined an axon-to-dendrite ratio (A:D ratio) [8]. To study the role of kinesin-1 we used a dominant negative comprising the cargo-binding domain of Kif5C that interferes with endogenous kinesin-cargo interactions fused to RFP (Kif5C-RFP-DN) [16]. The axonal targeting of MYC-GABA_B_R1a was markedly reduced in the presence of Kif5C-RFP-DN (Fig. 5A and Fig. S3, arrows. A:D ratio MYC-GABA_B_R1a 0.63±0.12; MYC-GABA_B_R1a plus Kif5C-RFP-DN 0.06±0.02; p<0.01). On the contrary, the axonal distribution of the ER marker ER-YFP and GABA_B_R2-EGFP were not affected by Kif5C-RFP-DN (Fig. 5B. A:D ratio ER-YFP 0.64±0.47; ER-YFP plus Kif5C-RFP-DN 0.63±0.32; p = 0.97; GABA_B_R2-EGFP 0.79±0.46; GABA_B_R2-EGFP plus Kif5C-RFP-DN 0.69±0.68; p = 0.80).

Kinesin-1 may control the axonal localization of MYC-GABA_B_R1a via an adaptor mechanism through the cytosolic C-terminal domain of GABA_B_R1a. To directly test this we first determined whether axonal targeting required the cytosolic C-terminal domain of GABA_B_R1a using two C-terminal subunit mutants, MYC-GABA_B_R1a-AA-ASA and MYC-GABA_B_R1a-ΔC [15]. GABA_B_R1a, GABA_B_R1a-AA-ASA and GABA_B_R1a-ΔC were all abundant in the axon indicating that targeting is independent of the C-terminal domain (Fig. 5C, arrows). Using a quantitative colocalization analysis based on Manders coefficients [31] all GABA_B_R1a constructs colocalized partially with the ER and no statistically significant difference was observed between GABA_B_R1a and the two mutants (MYC-GABA_B_R1a, n = 9 neurons; MYC-GABA_B_R1a-AA-ASA p = 0.11, n = 5 neurons; MYC-GABA_B_R1a-ΔC p = 0.30, n = 5 neurons). These results suggest that the C-terminal domain is not a major determinant of axonal ER distribution and targeting of GABA_B_R1a.

Finally, we analyzed the kinesin-1 dependence on the axonal targeting of GABA_B_R1a lacking the C-terminal domain. Axonal localization of MYC-GABA_B_R1a-ΔC was still severely impaired by the dominant negative construct (Fig. 5D, arrows. A:D ratio MYC-GABA_B_R1a-ΔC 0.42±0.06; MYC-GABA_B_R1a-ΔC plus Kif5C-RFP-DN 0.05±0.01; p<0.01). Combined these experiments demonstrate that kinesin-1 is necessary for the ER axonal targeting and localization of GABA_B_R1a. In addition, they indicate that the C-terminal domain of GABA_B_R1a, which is exposed to the cytosol, is not involved in this transport mechanism, further supporting the role of luminal or ER membrane domains in axonal sorting and targeting.

## Discussion

We have shown that GABA_B_Rs require the Golgi apparatus for plasma membrane delivery. Importantly, to our knowledge we have demonstrated for the first time that the sorting and targeting of an axonal neurotransmitter receptor, namely the GABA_B_R1a subunit, occur in a pre-Golgi compartment. Consistent with these observations, our evidence points to the fact that GABA_B_R1a traffics along the axonal ER and the ERGIC, and is transported by the molecular motor kinesin-1.

### Pre-Golgi sorting and targeting of axonal GABA_B_R1a

Our study indicates that axonal sorting of GABA_B_R1 subunits operates in the ER. As reported elsewhere, axonal targeting of GABA_B_R1a is unaltered in GABA_B_R2 knock-out neurons [8]. Additionally, the sushi domains located at the N-terminus of GABA_B_R1a, are sufficient for targeting even when placed in a non-related CD8α protein context [8]. Combined with the results presented here these observations imply that sorting and targeting signals exposed to the ER lumen mediate the axonal localization of GABA_B_R1a.

Historically, sorting of plasma membrane proteins to distinct membrane domains has been thought to occur exclusively at the Golgi or the trans-Golgi network, but accumulated evidence now favors the view that decisions are made at almost every step along the secretory pathway including the ER [32]. For example, mutations in Sec24p, a component of the coat protein complex II (COPII), selectively disrupt recruitment of cargo for ER to Golgi transport [33]. Likewise, synthetic cell penetrating peptides based on the cytosolic domain of the temperature-sensitive VSVG protein only inhibits the transport of a subset of cargo from the ER to the Golgi apparatus [34].

Two possible targeting mechanisms are conceivable for GABA_B_R1a in axons: (i) a luminal or membrane spanning ER protein enriched in the axonal ER subcompartment may bind the sushi domains and produce the accumulation of GABA_B_R1a but not GABA_B_R1b, in the axon (selective retention); or (ii) a protein or protein complex that spans the ER membrane may function as an adaptor between the GABA_B_R1a subunit and a molecular motor and selectively direct the transport of the subunits to axons (selective transport). Additional axonal scaffolding proteins may anchor the GABA_B_R1a subunit to strengthen axonal localization.

Although we cannot rule out the first alternative our data are consistent with a selective transport mechanism. Axonal targeting of GABA_B_R1a is not altered by its cytosolic C-terminal. Thus, transport may be controlled by a specific GABA_B_R1a N-terminal adaptor or by a general ER adaptor complex. Identification of these molecules in future studies is needed to strengthen this hypothesis. A mechanism compatible with (i) has been described for the rotavirus VP7 glycoprotein, which is retained in the ER. VP7 is still transported to the axon after BFA treatment [35]. Similar to GABA_B_R1a, VP7 uses a Golgi-independent intracellular sorting mechanism to reach the axon. One may envision the participation of chaperones in ER transport and targeting. For example, Hsc70 may provide a mechanism to release kinesin from cargo in specific subcellular domains, thereby producing the delivery of axonally transported cargo [36].

### Kinesin-1-dependent ER transport of GABA_B_R1a in axons

Since GABA_B_R1 is transported along the ER and has a limited residency period within the organelle under physiological conditions we refer to it as an ER-boarded protein. The precise transport mechanism of ER-boarded GABA_B_R1a in the axon is not clear. A kinesin-1-dependent transport of ER proteins has been described in dendrites [37]. Likewise, a microtubule-dependent transport of two ER resident proteins in the axon, GFP-SERCA and GFP-IP_3_R, has been observed in cultured chick dorsal root ganglion neurons [38]. Their bidirectionality and average velocities (∼ 0.1 µm/s) suggest that the transport is likely non-vesicular, and may represent lateral displacement within the continuous axonal ER membrane or mobility of the organelle itself. Directionality at steady state and average velocities observed for GFP-SERCA and GFP-IP_3_R range between slow (0.001–0.03 µm/s) and fast axonal transport (∼ 1 µm/s) [38], [39]. These intermediate velocities are conserved in our study suggesting that similar mechanisms may operate for the transport of ER-boarded GABA_B_R1a in axons. A conserved transport mechanism is also supported by the microtubule dependence of axonal GFP-SERCA, GFP-IP_3_R and GABA_B_R1a mobility. Thus, as reported for ER resident proteins al least one component of the mobility of GABA_B_R1a is not mediated by simple diffusion. However, precisely how the microtubule-cytoskeleton mediates the movement of integral membrane proteins within the continuous ER network is still unclear. According to the mechanisms originally put forward by Tsukita and Ishikawa [40], Waterman-Storer and Salmon [41] and others [42] kinesin-1 may control the lateral displacement of the subunit along the ER membrane in a conveyor-like system or mediate the microtubule-dependent ER sliding of the organelle itself, which may contribute to GABA_B_R1a transport. Since components of both mechanisms are dependent on kinesin-1 and stable microtubules it is difficult to discriminate between them. Because in this study we wanted to eliminate microtubules that serve as tracks to kinesin-1, the concentrations of nocodazole used were ∼15 fold higher than those that affect exclusively the population of dynamic microtubules (100 µM versus ∼ 6 µM). Thus, additionally, we cannot rule out the contribution of dynamic microtubules to the transport of GABA_B_R1a in the axon [41]. The existence of discrete mobile packets for axonal GABA_B_R1a also raises the possibility that these are not continuous with the ER network. Electron microscope studies in central and peripheral axons have revealed that the structure of the ER is predominantly contiguous but that it contains occasional free elements [40], [43]. Additionally, isolated and mobile compartments have been observed with fluorescent reporters [38]. Puncta-like behavior may also be explained by transitory tubule fission/fusion events [40] or lateral mobility of protein aggregates. The precise relationship between the mobile structures observed in this and other studies with the continuous ER network requires further studies. Nonetheless, our results support a kinesin-1-mediated axonal transport of ER-boarded GABA_B_R1a that is in agreement with previous reports.

The majority of the axonal GABA_B_R1a subunit was static during the live imaging conditions and intervals examined in this study. Additionally, the mobile fraction was higher for a mutant subunit that escapes the ER (MYC-GABA_B_R1a-EGFP: 19% mobile puncta, n = 259; MYC-GABA_B_R1a-ASA-AA-EGFP 33% mobile puncta, n = 247). It will be interesting to understand the significance and properties of the static component of GABA_B_R1a in the ER, and whether ER immobility plays a role in processing or trafficking.

Previous studies have demonstrated that GABA_B_Rs are segregated intracellularly and that blockade of ER exit results in the accumulation of heteromers in the soma and dendrites of hippocampal neurons [4]. These results suggest that GABA_B_R subunits are transported into dendrites independently and not as assembled heteromers. They also suggest that newly synthesized GABA_B_Rs assemble in the ER and exit throughout the somatodendritic compartment prior to insertion at the plasma membrane. This idea is in agreement with transport within the ER and disagrees with a long-haul post-Golgi vesicular transport of GABA_B_Rs, suggesting a non-canonical trafficking modality for GABA_B_Rs in dendrites. Thus, long-range ER transport may underlie both dendritic and axonal targeting of GABA_B_Rs. Since the local complexity of the ER network influences intracellular trafficking it may be utilized by neurons to control the availability of dendritic and axonal membrane proteins that are spatially restricted [44], [45].

### Axonal trafficking and ER/ERGIC recycling

In central and peripheral axons the ER is a continuous three-dimensional network of irregular tubules and cisternae [40], [43]. As mentioned above, several studies have demonstrated the localization of ER resident proteins and components of the protein folding and export machineries in the axon [19]–[20]. More importantly, the discovery of local assembly of COPII components in the axon supports the existence of functional ER exit sites required to process newly synthesized proteins that contribute to axonal outgrowth during the early stages of development [46]. However, direct evidence for local protein trafficking within early biosynthetic organelles in the axon is still lacking. In *Drosophila,* polarized secretion of the EGFR ligand from photoreceptor neurons includes local processing and secretion in the axon, and both mechanisms are controlled by ER localization [47].

More functional evidence is needed to conclusively demonstrate the role of a local trafficking pathway for GABA_B_R1a in axons. However, a fraction of GABA_B_R1a colocalizes and moves in synchrony with the ERGIC. This indicates that a local route involving export and retrieval between early biosynthetic organelles may contribute to GABA_B_R1a trafficking in the axon. A coat protein I complex (COPI) dependent retrieval mechanism from the cis-Golgi to the ER has been postulated for GABA_B_R1a [48], [49]. Whether this occurs in the axon and whether the remaining secretory steps operate locally to deliver GABA_B_R1a to the axonal plasma membrane remains unclear. In any case, the presence of the same RXR-type ER retention motif in GABA_B_R1a and GABA_B_R1b suggests that despite determining a rate-limiting step for ER exit, dwell time in the ER is not a major determinant of spatial range and axonal localization of GABA_B_R1a [50].

Overall our study demonstrates that ER sorting and local transport are relevant for axonal GABA_B_R trafficking. It is of great interest to determine to what extent the axonal ER is involved in the trafficking of other neurotransmitter receptors and ion channels that travel long distances, especially in long peripheral nerves.

## Supporting Information

Figure S1 **Antibodies recognize GABA_B_R1 subunits specifically.** (**A**) Increasing concentrations of lysates prepared from MYC-GABA_B_R1a transfected cells were applied on a nitrocellulose membrane and immunoblotted with control serum (control) or serum from a rabbit immunized with a GST fusion protein containing the C-terminal domain of GABA_B_R1 (αR1). (**B**) Lysates prepared from crude rat brain membranes (Brain), untransfected COS7 cells (Mock), or MYC-GABA_B_R1a transfected COS7 cells (MYC-GB1a) were immunoblotted with affinity purified GABA_B_R1 antibodies. (**C**) COS7 were transfected with MYC-GABA_B_R1a (MYC-GB1a, top), HA-GABA_B_R2 (HA-GB2, middle) or left untransfected (bottom). Cells were fixed and processed for immunofluorescence using MYC antibodies (green), affinity purified GABA_B_R1 antibodies (red) and HA antibodies (magenta). Merged images are shown on the right. Scale bar represents 20 μm.
(TIF)

Figure S2 **Recombinant GABA_B_R1a is retained in intracellular compartments in hippocampal neurons.** (**A**) Hippocampal neurons were transfected with MYC-GABA_B_R1a and processed for immunofluorescence under non-permeabilized conditions to detect cell surface epitopes followed by permeabilization to detect intracellular epitopes at the indicated days post-transfection (dpt). Intracellular MAP2 (red), intracellular GABA_B_R1a (MYC-GB1 intracellular, green), plasma membrane GABA_B_R1a (MYC-GB1 membrane, magenta). Control neurons were transfected with MYC-GABA_B_R1a and FLAG-GABA_B_R2 (+FLAG-GB2, right column). Merged images are shown on the bottom panel. (**B**) Axons of hippocampal neurons under the same experimental conditions. Images are not single focus planes, therefore the intensity represents the signal from the entire cell (representative images of n = 30 neurons). Scale bar for A–B represents 20 μm.
(TIF)

Figure S3 **Axonal targeting of GABA_B_R1a is kinesin-1 dependent.** (**A**) Hippocampal neurons were transfected with MYC-GABA_B_R1a (MYC-GB1) and RFP. Merged images are shown on the bottom panel. (**B**) Same as above for MYC-GABA_B_R1a and Kif5C-RFP-DN. Axonal localization of MYC-GABA_B_R1a or its absence from the axon is indicated by arrows. Scale bar represents 20 μm.
(TIF)
