# Supplementary figures and images for: Endoplasmic Reticulum Sorting and Kinesin-1 Command the Targeting of Axonal GABAB Receptors
Source: PLoS One. 2012 Aug 27;7(8):e44168. doi: 10.1371/journal.pone.0044168 (PMC3428321; doi:10.1371/journal.pone.0044168)

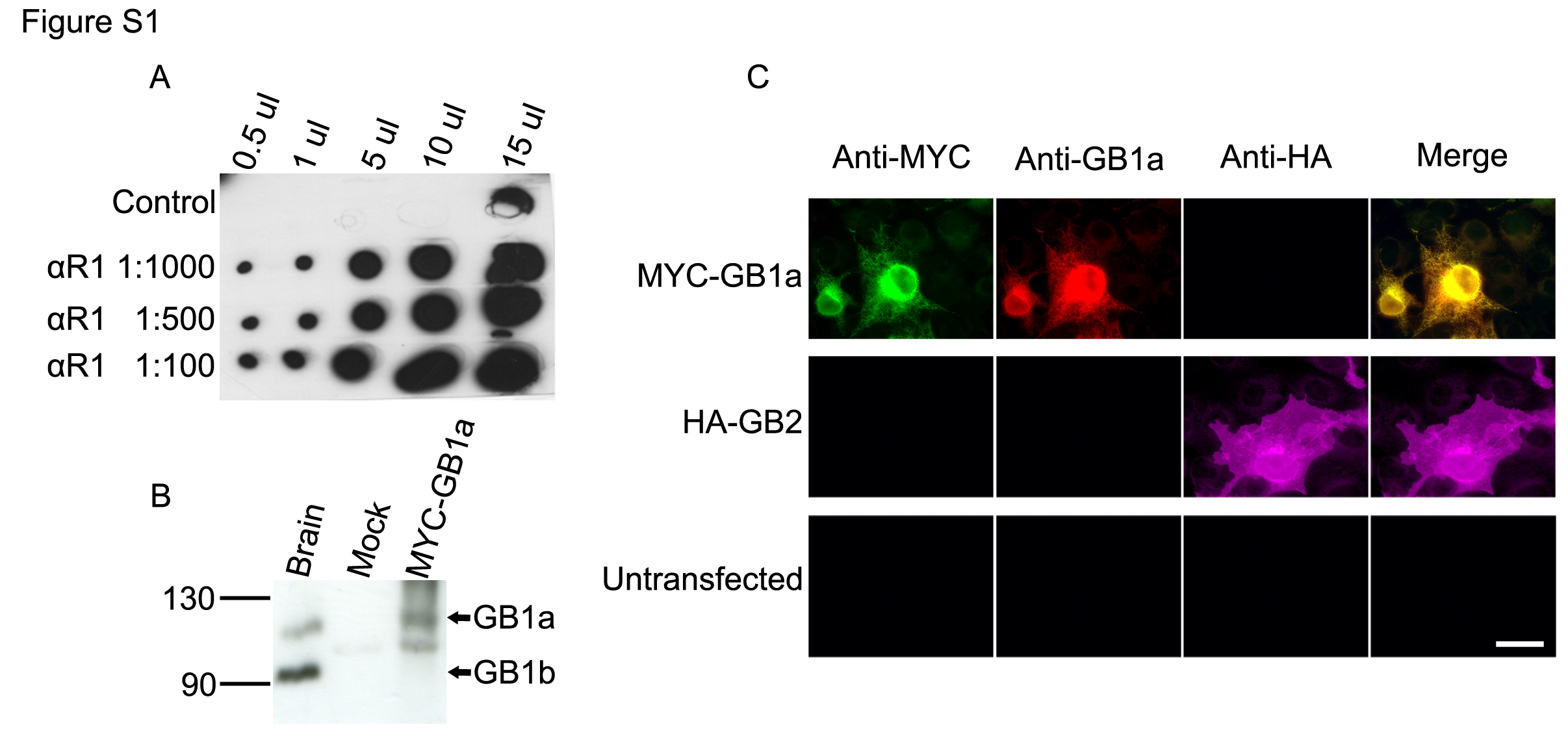

Supplement: Figure S1 — Antibodies recognize GABABR1 subunits specifically. (A) Increasing concentrations of lysates prepared from MYC-GABABR1a transfected cells were applied on a nitrocellulose membrane and immunoblotted with control serum (control) or serum from a rabbit immunized with a GST fusion protein containing the C-terminal domain of GABABR1 (αR1). (B) Lysates prepared from crude rat brain membranes (Brain), untransfected COS7 cells (Mock), or MYC-GABABR1a transfected COS7 cells (MYC-GB1a) were immunoblotted with affinity purified GABABR1 antibodies. (C) COS7 were transfected with MYC-GABABR1a (MYC-GB1a, top), HA-GABABR2 (HA-GB2, middle) or left untransfected (bottom). Cells were fixed and processed for immunofluorescence using MYC antibodies (green), affinity purified GABABR1 antibodies (red) and HA antibodies (magenta). Merged images are shown on the right. Scale bar represents 20 μm. (TIF) [file pone.0044168.s001.tif]

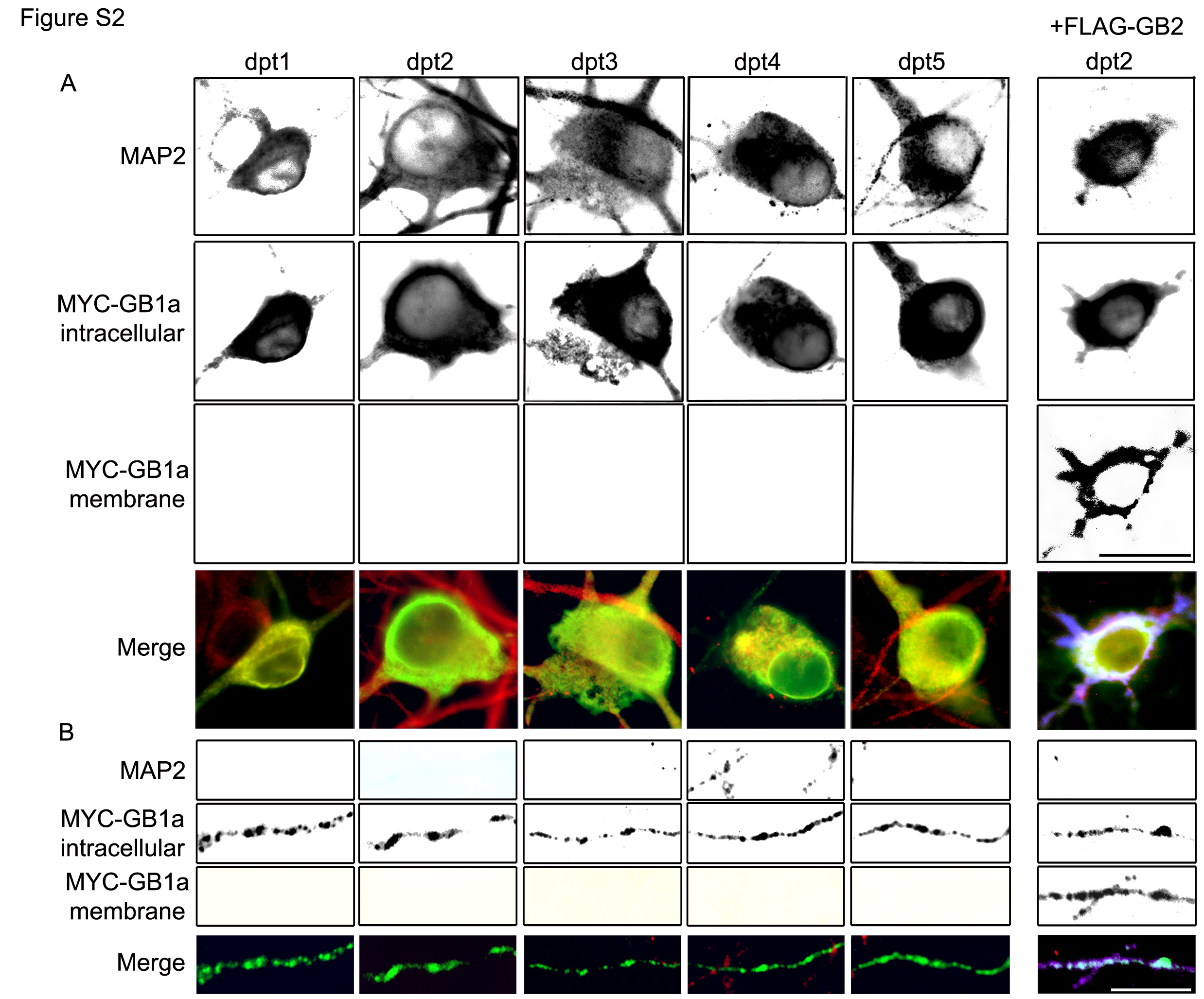

Supplement: Figure S2 — Recombinant GABABR1a is retained in intracellular compartments in hippocampal neurons. (A) Hippocampal neurons were transfected with MYC-GABABR1a and processed for immunofluorescence under non-permeabilized conditions to detect cell surface epitopes followed by permeabilization to detect intracellular epitopes at the indicated days post-transfection (dpt). Intracellular MAP2 (red), intracellular GABABR1a (MYC-GB1 intracellular, green), plasma membrane GABABR1a (MYC-GB1 membrane, magenta). Control neurons were transfected with MYC-GABABR1a and FLAG-GABABR2 (+FLAG-GB2, right column). Merged images are shown on the bottom panel. (B) Axons of hippocampal neurons under the same experimental conditions. Images are not single focus planes, therefore the intensity represents the signal from the entire cell (representative images of n = 30 neurons). Scale bar for A–B represents 20 μm. (TIF) [file pone.0044168.s002.tif]

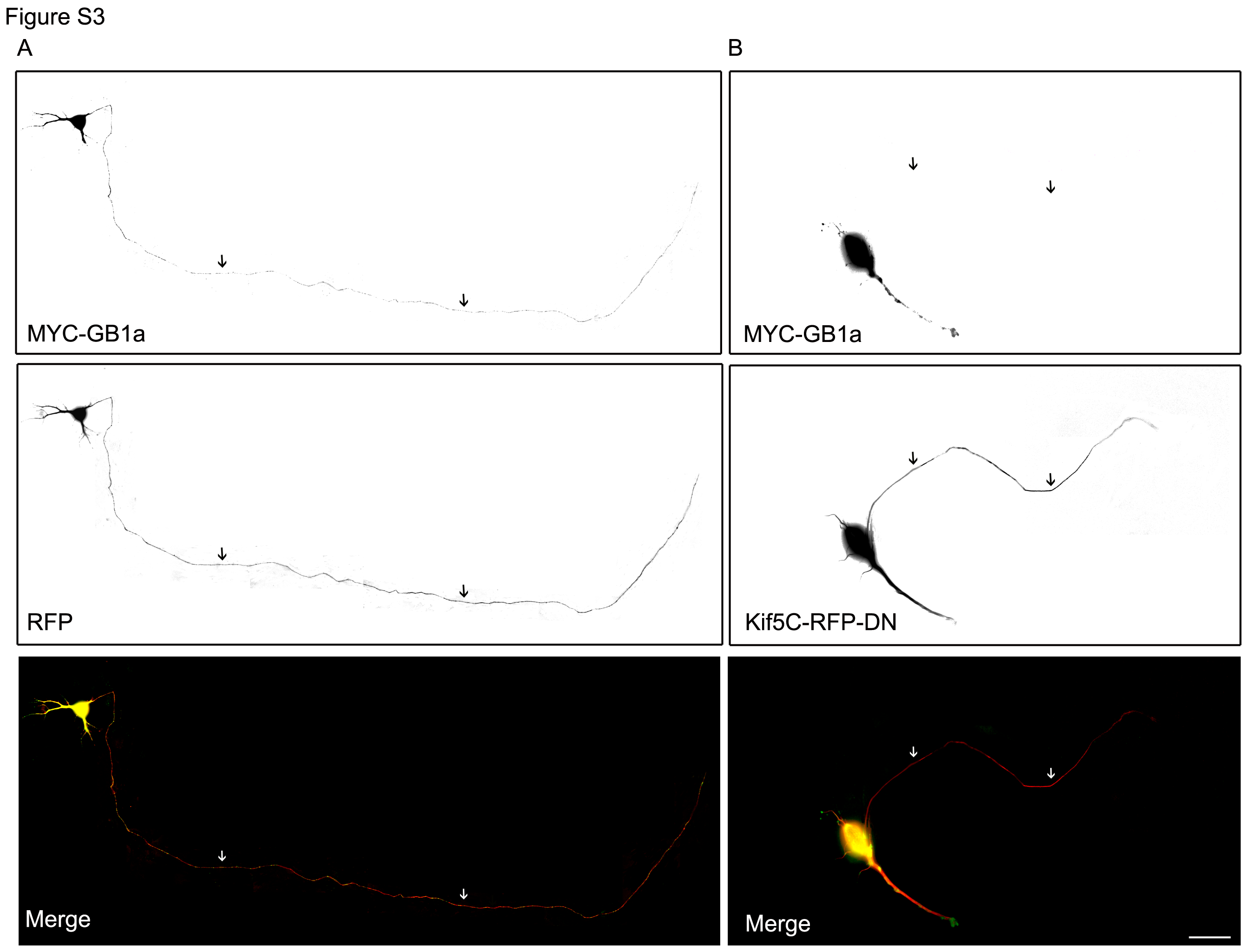

Supplement: Figure S3 — Axonal targeting of GABABR1a is kinesin-1 dependent. (A) Hippocampal neurons were transfected with MYC-GABABR1a (MYC-GB1) and RFP. Merged images are shown on the bottom panel. (B) Same as above for MYC-GABABR1a and Kif5C-RFP-DN. Axonal localization of MYC-GABABR1a or its absence from the axon is indicated by arrows. Scale bar represents 20 μm. (TIF) [file pone.0044168.s003.tif]
